# Complete Genome Sequence of the Last Representative Genotype of Wild Indigenous Poliovirus Type 1, Which Circulated in Brazil

**DOI:** 10.1128/genomeA.00811-13

**Published:** 2013-10-31

**Authors:** Fernando N. Tavares, Eliane V. da Costa, Olen M. Kew, Edson E. da Silva

**Affiliations:** Laboratório de Enterovírus, Instituto Oswaldo Cruz, FIOCRUZ, Rio de Janeiro, Rio de Janeiro, Brazila; Laboratório de Enterovírus, Seção de Virologia, Instituto Evandro Chagas/IEC, Ananindeua, Pará, Brazilb; Centers for Disease Control and Prevention, National Center for Immunization and Respiratory Diseases, Division of Viral Diseases, Atlanta, Georgia, USAc

## Abstract

Polioviruses are the major etiological agents associated with acute flaccid paralysis (AFP). The complete genome sequence of a representative of the last wild poliovirus type 1 genotype isolated in Brazil from a paralytic poliomyelitis case is reported here.

## GENOME ANNOUNCEMENT

Polioviruses belong to the genus *Enterovirus*, family *Picornaviridae*, are among the most important and well-studied human pathogens, and are the etiologic agents of paralytic poliomyelitis (1, 2).

Although the oral poliovirus vaccine (OPV) was introduced into the routine immunization program for infants in Brazil in the early 1960s, the circulation of wild poliovirus dropped sharply only after the implementation of National Immunization Days (NIDs) in 1980, which set the goal of vaccinating in a single day, twice a year, the entire population of children <5 years of age, irrespective of their vaccination history (3, 4). The incidence of poliomyelitis dropped from an average of 2,330 cases in the 1975-1980 period to only 122 cases in 1981. The last case of paralytic poliomyelitis caused by endemic transmission of wild poliovirus occurred in March 1989 in the northeast region of the country (5).

Here, we report a genomic study of the last representative genotype of wild poliovirus type 1 that circulated in Brazil through complete genome sequencing. The virus was originally isolated from fecal specimens from a patient with acute flaccid paralysis in 1988. Viral RNA was extracted from the cell supernatant of RD cells and cDNA synthesized with oligo(dT) primer. Amplifications were done using internal primer pairs to generate overlapping amplicons spanning the entire viral genome, while the 3′- and 5′-end sequences were determined using Race kits (Life Technologies). Sequencing reactions were analyzed in an ABI 3130xl genetic analyzer, and contigs were assembled using SeqMan version 7.0 (DNAStar Lasergene, USA).

The genome of poliovirus 1 (PV1) strain 558 is 7,445 nucleotides (nt) in length. The alignments of strain 558 with the prototype wild PV1 Mahoney strain and Sabin 1 strain LSc-2ab were performed using ClustalX 2.1 (6). Strain 558 shares 81.6% and 81.4% nucleotide homology with the Mahoney and Sabin 1 genomes, respectively. The 5′-noncoding (NC) region is 747 nucleotides in length, and five nucleotide insertions (relative to Mahoney) are located at positions 101, 102, 117, 385, and 577. Throughout the coding segment (6,627 nt/2,209 amino acids [aa]), we found 66 and 69 amino acid differences from Mahoney/Sabin 1, respectively, distributed as follows: VP4, 1/2; VP2, 4/4; VP3, 1/2; VP1, 19/19; 2A, 7/6; 2B, 11/10; 2C, 6/6; 3A, 3/2; 3B, 0/1; 3C, 6/6; and 3D, 8/11. No insertions or deletions were observed inside the coding region.

Phylogenetic analysis based on the 906 nucleotides that encode the major capsid protein (VP1) was conducted using MEGA5.0 (7) software and showed that PV1 isolate 558 is closely related to a wild PV1 from Argentina/1982 (accession no. AF528783), sharing 88.0% nucleotide homology.

### Nucleotide sequence accession number. 

The complete genome sequence of PV1 isolate 558 has been deposited in GenBank under the accession no. KF537633.
